# Synthesis of Reduced Graphene Oxide with Adjustable Microstructure Using Regioselective Reduction in the Melt of Boric Acid: Relationship Between Structural Properties and Electrochemical Performance

**DOI:** 10.3390/nano8110889

**Published:** 2018-11-01

**Authors:** Justina Gaidukevič, Rasa Pauliukaitė, Gediminas Niaura, Ieva Matulaitienė, Olga Opuchovič, Aneta Radzevič, Gvidas Astromskas, Virginijus Bukauskas, Jurgis Barkauskas

**Affiliations:** 1 Faculty of Chemistry and Geosciences, Vilnius University, Naugarduko str. 24, LT-03225 Vilnius, Lithuania; justina.gaidukevic@chf.vu.lt (J.G.); pauliukaite@ftmc.lt (R.P.); olga.opuchovic@chf.vu.lt (O.O.); jurgis.barkauskas@chf.vu.lt (J.B.); 2 Department of Nanoengineering, Center for Physical Sciences and Technology, Savanorių Ave. 231, LT-02300 Vilnius, Lithuania; aneta.radzevic@ftmc.lt; 3 Department of Organic Chemistry, Center for Physical Sciences and Technology, Saulėtekio Ave. 3, LT-10257 Vilnius, Lithuania; ieva.matulaitiene@ftmc.lt; 4 Department of Physical Technologies, Center for Physical Sciences and Technology, Saulėtekio Ave. 3, LT-10257 Vilnius, Lithuania; astromskas@ftmc.lt (G.A.); virginijus.bukauskas@ftmc.lt (V.B.)

**Keywords:** graphene oxide, adjustable microstructure, boric acid, regioselective catalyst, oxygen scavenger

## Abstract

The melt of H_3_BO_3_ was used to reach a controllable reduced graphene oxide (rGO) synthesis protocol using a graphene oxide (GO) precursor. Thermogravimetric analysis and differential scanning calorimetry (TG/DSC) investigation and scanning electron microscopy (SEM) images have shown that different from GO powder, reduction of GO in the melt of H_3_BO_3_ leads to the formation of less disordered structure of basal graphene planes. Threefold coordinated boron atom acts as a scavenger of oxygen atoms during the process of GO reduction. Fourier-transform infrared (FTIR) spectra of synthesized products have shown that the complex of glycerol and H_3_BO_3_ acts as a regioselective catalyst in epoxide ring-opening reaction and suppress the formation of ketone C=O functional groups at vacancy sites. Thermal treatment at 800 °C leads to the increased concentration of point defects in the backbone structure of rGO. Synthesized materials were tested electrochemically. The electrochemical performance of these materials essentially differs depending on the preparation protocol. The highest charge/discharge rate and double-layer capacitance were found for a sample synthesized in the melt of H_3_BO_3_ in the presence of glycerol and treated at 800 °C. The effect of optimal porosity and high electrical conductivity on the electrochemical performance of prepared materials also were studied.

## 1. Introduction

Graphene oxide (GO) is widely used in energy, optical, electronic and sensor devices as a precursor of graphene and reduced GO (rGO) [1,2,3]. GO can be effectively prepared by chemical methods in large quantities. It possesses different types of oxygen functionalities which allows GO to be easily dispersed in organic and inorganic solvents to produce the films and coatings [2,4]. The process of reconstruction of π-conjugated graphene structure from GO is of particular importance for the quality of graphene-based devices. Methods used for the repair of graphene structure are based on the reduction of GO and removal of oxygen functionalities. Despite the recent progress in this area, a controlled reconstruction of graphene structure from GO precursor along with nanoscale engineering is still a problem. 

The thermal reduction of GO by high temperature annealing is highly efficient [5]. However, extensive release of gaseous products during the thermal treatment is difficult to control; it leads to the extensive disruption of graphene network [6]. Moreover, GO is capable of self-heating and spontaneous decomposition explosive scenarios [7]. With the expectation of controlled rGO synthesis, novel chemical strategies need to be found to scavenge the oxygen atoms before the vigorous decomposition reaction is started. A better control over surface chemistry and structure of rGO is the focus of many researcher efforts, who aim to repair the graphene structure by chemical reduction of GO and subsequent removal of oxygen functionalities [8,9]. 

Several methods for the non-oxidative intercalation and exfoliation of GO have been proposed to avoid the damage of the basal planes of graphene by using Brønsted acids (phosphoric, sulfuric, dichloroacetic, alkylsulfonic, etc.) [10]. A viable option for non-oxidative exfoliation should be the use of orthoboric acid-H_3_BO_3_ (BA), as the planar structure of H_3_BO_3_ molecule should be beneficial for the intercalation between GO layers [11]. Although the orthoboric acid is a weak Brønsted acid, in the media of anhydrous sulfuric acid the H_3_BO_3_ is classified as a superacid [12]. Moreover, the process of GO reduction in the melt of H_3_BO_3_ is of great interest, since the boron atom is regarded as essentially electrophilic in character [13],, while the basal plane of graphene is a nucleophile (i.e., a region of increased electron density above and below the ring plane) [14]. Advances of regioselectivity open the possibility of applying these reactions for structural design of rGO with adjustable porosity. Niu et al. have effectively used BA as the precursor to produce the boron-doped electrode material for supercapacitor with outstanding electrochemical performance [15]. The aim of this research is to verify the efficiency of H_3_BO_3_ for controlled reduction of GO as well as find the optimal conditions for the reduction of GO used in energy, optical, electronic and sensor devices.

## 2. Materials and Methods

### 2.1. Synthesis of GO

GO was synthesized from the natural graphite according to the protocol reported by Yan et al. [16]. In a typical experiment, graphite powder (99.5%, Merck, Darmstadt, Germany) was treated with conc. H_2_SO_4_ (≥98%, Eurochemicals, Bratislava, Slovakia), K_2_S_2_O_8_ (99.99%, Sigma-Aldrich, Darmstadt, Germany) and P_2_O_5_ (≥99%, Sigma-Aldrich, Darmstadt, Germany). Then, this pre-oxidized graphite was subjected to oxidation by Hummers method [17]. Afterwards the GO particles were washed several times with distilled water by centrifugation (ultracentrifuge SIGMA 1-6P, Osterode am Harz, Germany) at 5500 rpm (each run for 20 min). The mixture was transferred into a dialysis tubing cellulose membrane with a cut-off molecular weight of 10,000–20,000 Da (Nadir^®^-dialysis tubing cellulose hydrate, Carl Roth GmbH, Karlsruhe, Germany) and dialyzed against distilled water until the dialysate was free of sulphate and exhibited a pH equal 6. The suspension was filtered using a Buchner funnel and obtained brown powder was dried in a vacuum desiccator to a constant weight.

### 2.2. GO Reduction in the Melt of Boric Acid

Synthesized GO was used as a precursor for the reduction in the melt of H_3_BO_3_ (≥99.5%, Sigma-Aldrich, Darmstadt, Germany) with/without addition of L-ascorbic acid (C_6_H_8_O_6_, 99%, Sigma-Aldrich, Darmstadt, Germany) and glycerol (C_3_H_8_O_3_, ≥99.5%, Sigma-Aldrich, Darmstadt, Germany). Ascorbic acid and glycerol are known to form a complex compound with H_3_BO_3_ [13,18,19]. Moreover, ascorbic acid is reported to be an efficient reducing agent for GO [19]. The summarized data of all synthesized samples are presented in the Supplementary data section (Table S1).

During the first stage, modified rGO samples (mrGO) were prepared. First, GO and H_3_BO_3_ (in a weight ratio 1:10) were grinded in an agate mortar for 10 min, transfused into a porcelain plate and placed into a furnace (SNOL 8.2/1100) (Utena, Lithuania) at 300 °C for 2 h and later at 400 °C for 6 h. At the beginning of the process dense bubbles emerged from the melt. The cooled reaction mixture was thoroughly washed with hot distilled water to remove the excess of H_3_BO_3_. The synthesized product was named GOBA (Table S1).

At the same time, the modification of GO with the mixture of H_3_BO_3_ and ascorbic acid or glycerol was carried out to synthesize a GOBA/AA and GOBA/G product, respectively. The synthesis protocol was the same as in the case of GOBA except that the GO was added to the mixture of H_3_BO_3_ and ascorbic acid or H_3_BO_3_ and glycerol in a weight ratio 2:20:1 (Table S1). The reduction of GO powder without addition of H_3_BO_3_ was carried out under the same conditions to synthesize GO/T as a baseline of mrGO.

### 2.3. Thermal Treatment of MrGO Samples

Prepared mrGO samples were used as precursors for the further modification by a thermal treatment (trGO samples). They were annealed in argon ambient at 800 °C for 1 h, the flow rate of argon was maintained 60 mL min^–1^ (Table S1). The synthesized products (GOBA/T, GOBA/AA/T and GOBA/G/T) as well as their precursors were used for the further investigations.

### 2.4. Materials Characterization

Thermogravimetry/differential scanning calorimetry (TG/DSC) analysis was carried out using a Thermal analyser STA6000 from PerkinElmer (Waltham, MA, USA). The analysis was performed with a heating rate of 2.5 °C min^–1^ (for mrGO samples) or 5 °C min^–1^ (for trGO samples) from room temperature to 400 or 800 °C under air (for mrGO) or N_2_ (for trGO) purge gas. 

FTIR spectra were recorded in transmission mode by using ALPHA spectrometer (Bruker, Inc., Bremen, Germany), equipped with a room temperature detector DLATGS. The spectral resolution was set at 4 cm^−1^. Spectra were acquired from 100 scans in the spectral range of 400–4000 cm^−1^. Samples were dispersed in KBr tablets. 

Raman spectra were recorded using inVia Raman spectrometer (Renishaw, Wotton-under-Edge, Gloucestershire, UK) equipped with thermoelectrically cooled (−70 °C) CCD camera and 1800 grooves mm^–1^ grating. The He-Ne gas laser provided an excitation beam at 632.8 nm. The laser power was restricted to 0.1 mW. The integration time was 800 s. Raman spectra were taken using a 20×/0.40 NA (Leica, Wetzlar, Germany) objective lens. The Raman frequencies were calibrated using the silicon standard according to the line at 520.7 cm^−1^. Frequencies and intensities of the Raman bands were determined by fitting the experimental contour with the Gaussian-Lorentzian form components by using GRAMS/AI 8.0 (Thermo Fisher Scientific Inc., Waltham, MA, USA) software. Correlation length (*L*_α_) was determined using the following equation [20]:

(1) $$L_{a}\left(nm\right)=(2.4\times 10^{-10)}\lambda_{L}^{4}{\left(\frac{A\left(D\right)}{A\left(G\right)}\right)}^{-1}$$

where *λ_L_* is laser excitation wavelength (nm) and *A(D)/A(G)* is the ratio of integrated intensities of D and G modes.

Scanning electron microscope (SEM) images were obtained using a Hitachi SU-70 microscope (Tokyo, Japan) at an accelerating voltage of 5.0 kV at magnifications of 25,000 and 50,000. 

The crystallographic information of the samples was characterized using a MiniFlex II (Rigaku, Tokyo, Japan) diffractometer with Cu Kα (Kα_1_ = 1.54056 Å) radiation. XRD of the powder samples was recorded for 2θ values from 5 to 55°. The characterization was done at 30 kV and 15 mA with a step size of 0.010° and a dwell time of 1.0 s. 

Electrical conductivity of the synthesized samples was determined using a cell prepared in the laboratory. The experiments were performed at room temperature (22 °C) and relative humidity (RH) of 40%. Approximately 0.03 g powder samples were placed in a 2.7 mm diameter glass cylinder between two metallic (Cu) electrodes. We consider, that the electrical contact area between the electrode and the powder was the same as the inner area of a cylinder. The sample was compressed gradually and the distance between electrodes was measured. The conductivity dependence on the bulk density was determined. A Keithley 2601 Source Meter (GlobalTech Sourcing, North Hampton, NH, USA) was used for the conductivity measurement. The resistivity (*ρ*) was determined using the following equation [21]:

(2) $$\rho =R\frac{A}{l}$$

where, *R* is a resistance (Ω), *A* is a cross section area (m^2^), *l* is length of the sample (m). The resistance of the samples was calculated from a slope of linear I-V characteristic curve (Figure S1). It is important to note, that I-V characteristic curves were linear in a voltage range of ±1 V for all investigated samples. 

The surface areas and pore volume distribution of the samples were measured by nitrogen adsorption isotherms at 77 K on a Brunauer–Emmett–Teller (BET) analyser TriStar II 3020, (Micromeritics, Norcross, GA, USA). Prior to the gas sorption measurements, all the samples were outgassed in N_2_ atmosphere at 120 °C for 2 h. Total surface areas S_BET_ were calculated using the Brunauer–Emmett–Teller model, whereas the t-plot method was used to estimate the micropores volume (*V*_μ_) and external surface area (of meso- and macropores) (*S*_EXT_). The total pore volume (*V*_tot_) was obtained from N_2_ amount adsorbed at a relative pressure close to unity.

### 2.5. Electrochemical Measurements

Cyclic voltammetry (CV) measurements were conducted with the CompactStat potentiostate/galvanostate with impedance module (Ivium Technologies, Eindhoven, The Netherlands). The three-electrode system was used employing the graphene oxide modified pyrolytic graphite (PGE) as a working electrode (diameter 3 mm), Pt wire was as a counter electrode and Ag/AgCl (KCl sat.) served as a reference. CVs at the modified PGE were recorded in the potential range from 0.0 to 1.0 V with the start potential at 0.0 V in 0.1 M K_2_SO_4_. Potential sweep rate was 0.1 V s^–1^. Neutral electrolyte K_2_SO_4_ was chosen, since such an electrolyte is more convenient for integration of possible supercapacitor with biosensors. The best neutral electrolyte for supercapacitors is indicated as sodium or potassium sulphate [22].

Charge/discharge curves were recorded with the same potentiostate but using different electrochemical cell composed from two symmetrical graphite plate electrodes and 10 µm thick polycarbonate membrane (Millipore Inc., Carrigtwohill, Ireland). 2.4 mg of GO material was weighted mixed with 50 µL of electrolyte and placed on the electrodes. The working area was 0.25 cm^2^. Two charge and discharge cycles were performed at 1.2 mA (0.5 A g^–1^) for 60 s each.

## 3. Results and Discussion

### 3.1. Investigation of the Reduction Process of GO in the Melt of Boric Acid

**Figure 1:**
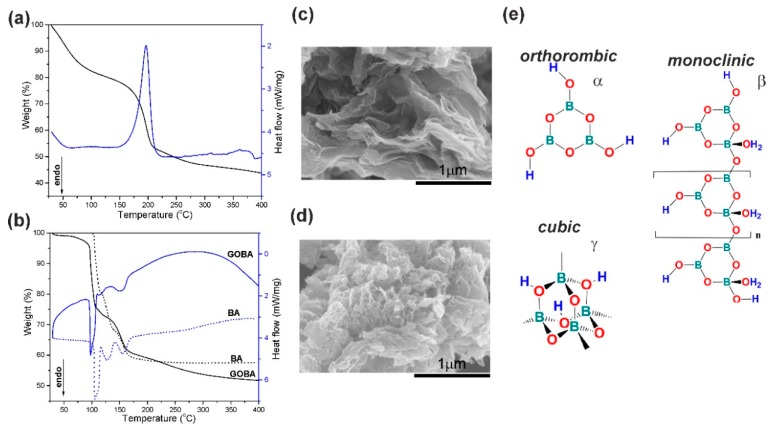
Thermogravimetric (TG) (black line)/differential scanning calorimetry (DSC) (blue line) thermograms obtained in the systems composed of boric acid (BA) and graphene oxide (GO): (**a**) pristine GO powder; (**b**) BA and precursor mixture of GOBA Scanning electron microscopy (SEM) images of rGO samples: (**c**) GOBA and (**d**) GO/T; (**e**) Representative structures of monotropic forms of HBO_2_.

A new method of GO reduction was conducted in the melt of BA. The changes occurring in the process of GO reduction were identified employing TG/DSC analysis. The TG/DSC thermograms of the precursors and reaction mixtures are depicted in Figure 1 and in the Supplementary data section (Figure S2).

In Figure 1a the TG/DSC thermograms of the thermal reduction of pure GO powder are presented. The weight loss up to 130 °C occurs due to the removal of adsorbed or intercalated water. Meanwhile, the second mass loss in the temperature range from 130 to 400 °C can be ascribed to the release of CO_2_ and CO from the decomposition of labile oxygen containing functional groups, which results in vigorous exothermic peak at 200 °C in the DSC curve [7,23,24].

The TG thermogram of BA (Figure 1b) indicates three rapid weight loss regions. The weight loss in the region 93–113 °C is due to the evaporation of water and H_3_BO_3_ molecules conversion into a metaboric acid HBO_2_, which results in endothermic peaks at 105 °C in the DSC curve. In the temperature range from 113 to 140 °C, transitions between the three monotropic forms of HBO_2_ (α-, β- and γ-) take place, which leads to an endothermic peak at 129 °C [25]. At stage three (140–210 °C), initial BA melt is turned into boron oxide B_2_O_3_, which results in endothermic peaks at 155 °C.

The TG curve of the precursor mixture of GOBA exhibits a similar trend like BA sample but do not coincide, indicating some differences of reduction process (Figure 1b). The weight loss of the precursor mixture of GOBA at the beginning is more significant in comparison with that of BA. Later, at ~105 °C, the water lost by GO is accumulated by BA. By comparing two DSC thermograms of BA and the precursor mixture of GOBA, similar characteristic peaks can be found but their positions and heights also indicate the differences between the both processes. The second DSC peak of the GOBA precursor thermogram is shifted from 129 °C to 114 °C and it has much lower intensity comparing to that of pure BA sample. This is due to the exothermic rearrangements in GO, which quench the endothermic transitions between monotropic α-, β- and γ- forms of HBO_2_.

The TG/DSC data show that the reduction of GO in the melt of BA consists of two parallel process. The first, that slowly occurs in a wide temperature range (from 30 to 400 °C), is due to removal of oxygen functional groups and the second one coincides with the monotropic transitions between α-, β- and γ-HBO_2_ (113–140 °C). Simultaneously, it was determined that in comparison to the thermal reduction of pure GO (Figure 1a), gradual reduction of GO in the melt of BA takes place even at moderate temperatures. This phenomenon was observed among others by Wang et al. [26], who found that BA could catalyse the dehydration and other oxygen-eliminating reactions of wood at a relatively low temperature (approximately 100 °C to 300 °C). 

Considering the reaction mechanism, the process of GO reduction in the melt of BA, as in all other cases, can be roughly divided into thermal and chemical pathways [5]. In the case of the GOBA synthesis, the thermal reduction may be identified with DSC peak, while the chemical reduction should occur in the region of monotropic transitions of HBO_2_ (113–140 °C). Moreover, the GO reduction in the melt of BA proceeds much more smoothly in comparison with GO powder. The morphology of both products (GO/T and GOBA) displays the different ways of GO reduction; GO/T sample is characterized by much more irregular stacking and extensive disruption of graphene layers in comparison with GOBA (Figure 1c,d). Evidently, the use of the melt of BA can be applied for controllable exfoliation of GO sheets.

In this connection, attention should be drawn to the fact that all three monotropic forms of metaboric acid can be characterized by different coordination number of boron atom: 3 for orthorhombic α-HBO_2_, 3 or 4 for monoclinic β-HBO_2_ and 4 for cubic γ-HBO_2_ (Figure 1e) [25]. During the change of coordination number, in the temperature range of 113–140 °C, the electrophilic character of HBO_2_ was reported to be the highest [13]. Moreover, according the literature, the basal plane of graphene (and, to some extent, that of GO) has an increased electron density [14]. Consequently, threefold coordinated boron atom as electrophile, the particle with a lack of electron density, should attack the basal plane of graphene. Namely, threefold coordinated boron atom in α-HBO_2_ can act as a scavenger for oxygen atoms during the process of GO reduction.

Ascorbic acid and glycerol were added to facilitate GO reduction in BA melt. The TG/DSC thermograms obtained after addition of ascorbic acid or glycerol to the reaction mixture are given in the Supplementary data section (Figure S2a,b). The reduction process was found to display similar routes as for the precursor mixture of GOBA. Two steps of mass loss were observed. The first loss in the temperature range of 70–140 °C is mainly ascribed to the removal of adsorbed water and transitions between the three monotropic forms of HBO_2_. The weight loss in temperature range of 140–400 °C can be assigned to the decomposition of organic group or labile oxygen functional groups in the material [23,27,28]. In addition, a very small difference is observed between the TG thermograms of BA/AA and the precursor mixture of GOBA/AA (Figure S2b). Supposedly, the reduction of GO with ascorbic acid occurs in the stage of grinding of the reaction mixture in an agate mortar before heating with BA and the difference between the TG curves is reduced to a minimum. Meantime, the glycerol is active in the heating stage (Figure S2a). The action of glycerol is explained below by comparing the TG/DSC and structural data.

Finally, the mrGO samples were subjected to the thermal treatment up to 800 °C (Figure S2c). The TG/DSC thermograms show that up to 400 °C no significant changes have been developed and no sharp transition peaks appear for all trGO samples. Apparently, the thermal treatment of GO in the BA melt leads to the formation of a thermodynamically stable structure. Above 400 °C all mrGO samples undergo further structural rearrangement accompanied by substantial weight loss. The most pronounced weight loss is for the GOBA/AA/T (45%).

### 3.2. Structural Characterization of GO Reduction Products

**Figure 2:**
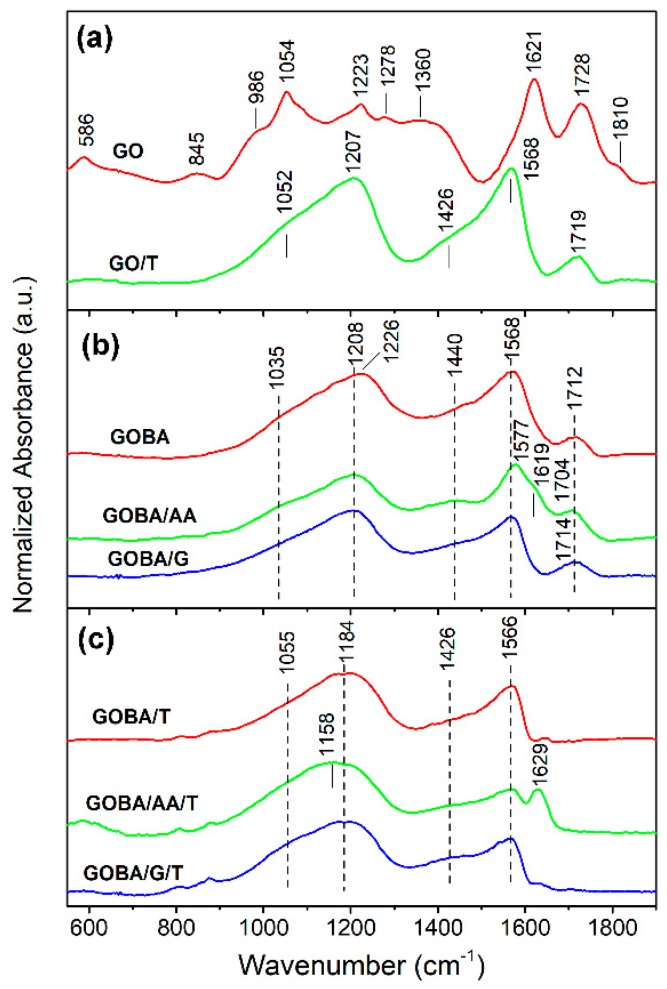
(**a**) Fourier transform infrared (FTIR) spectra of pristine GO and GO/T; (**b**) FTIR spectra of mrGO samples; (**c**) FTIR spectra of trGO samples.

Infrared spectroscopy is useful to provide insights into the state of particular functional groups during the GO reduction process [29,30,31,32,33,34,35,36]. Figure 2 compares FTIR spectra of GO reduction products and their precursor. The FTIR bands are assigned to definite vibrations of functional groups.

Infrared spectrum of sample GO/T (Figure 2a) shows that the thermal reduction of graphene oxide powder results in disappearance of epoxide (845 and 1278 cm^−1^), ketone (1621 cm^−1^), non-conjugated ketone (1810 cm^−1^) and hydroxyl group (1360 cm^−1^) bands; while absorption of C−O bonds (900–1300 cm^−1^) and carboxyl C=O groups (1728 cm^−1^) considerably decreases. A strong band of GO/T sample near 1568 cm^−1^ was assigned to in-plane C=C asymmetric stretching vibration of aromatic rings of sp^2^ hybridized carbon [29,32]. Intensity of this band increases after the removal of out-of-plane species (epoxide and hydroxyl groups) [27]. Remains of some C−O and C=O bonds are visible from the presence of absorption bands at 1052−1207 and 1719 cm^−1^, respectively. The shoulder near 1426 cm^−1^ corresponds to bending vibration of OH group δ(OH), as was recently predicted by DFT calculations coupled with infrared spectroscopy studies [32]. Theoretical analysis has demonstrated that these OH groups are closely associated with the defects in basal graphene plane.

The comparison spectra of GOBA, GOBA/AA and GOBA/G samples (Figure 2b) shows that the most intense δ(OH) band remains for GOBA/AA. The clear band visible near 1440 cm^−1^ in the spectrum of sample GOBA/AA indicates presence surface OH groups after reduction process. In addition, existence of ketone groups at vacancy sites in this sample is visible from the shoulder near 1619 cm^−1^ [32]. Finally, thermal treatment of the samples results in complete elimination of C=O stretching bands in the frequency range of 1704−1714 cm^−1^ (Figure 2c). However, contrary to GOBA/T and GOBA/G/T samples, clear absorption band of ketone groups at vacancy sites is visible near 1629 cm^−1^ in the case of GOBA/AA/T. 

**Figure 3:**
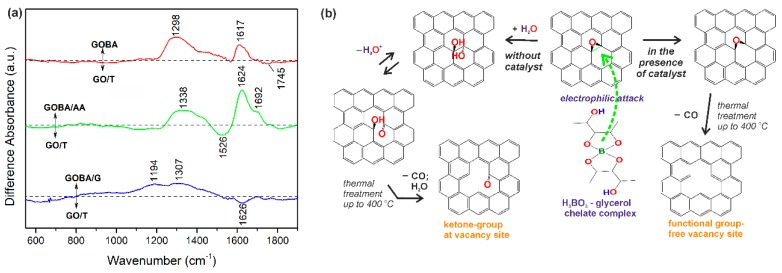
FTIR-difference spectra of mrGO samples. (**a**) Spectra are normalized according to absorbance of C=C stretching band of sp^2^ hybridized carbon at 1568 cm^−1^; (**b**) Scheme of GO reduction reactions occurring in the presence and absence of BA-glycerol chelate complex catalyst.

To improve visualization by comparing mrGO samples with GO/T, FTIR-difference spectra have been constructed (Figure 3a). The analysis of FTIR-difference spectra along with the data from Figure 2 evidence that ketone groups at vacancy sites are clearly visible in both GOBA and, especially, GOBA/AA samples (positive-going bands at 1617−1624 cm^−1^). However, relative amount of ketone C=O groups is lower in the case of GOBA/G sample comparing with GO/T one (negative-going peak at 1626 cm^−1^). Such an effect of glycerol addition should be due to the activity of BA-glycerol complex, which is known as a regioselective catalyst for epoxide ring-opening reactions [18]. 

Based on the results obtained by the FTIR analysis, representative reaction scheme was proposed for operation of BA-glycerol chelate complex for epoxide ring-opening reaction (Figure 3b). Due to increased electron density on the basal plane of GO [19], the GO surface is attacked by an electrophilic BA-glycerol complex. Following the reaction scheme shown in Figure 3b, the regioselective BA-glycerol chelate complex catalyst is able to suppress the formation of –OH functional groups that may originate from epoxide ring-opening [4]. Moreover, it is able to suspend the formation of C=O functional groups at vacancy sites during thermal treatment up to 400 °C. Meanwhile, in the case of GO reduction without catalyst, the epoxide ring-opening reaction occurs on the basal plane and is followed by the thermal treatment up to 400 °C. In addition, when the reaction occurs without the catalyst, the ketone C=O groups are formed at the vacancy sites (Figure 3b). These two reaction paths are also validated on the base of the model proposed by Dimiev and co-authors [37]. 

Raman spectroscopy was also performed because it provides detailed information on structure of carbon network in graphene-based materials. The results of Raman analysis are shown in Supplementary data section (Figure S3) and Table 1. The results obtained (Table 1) show that the D-band position shifts to lower frequencies after the thermal treatment of mrGO (to 1330 cm^−1^). Such shift indicates structural point defects emerge at high temperatures [38]. The increase in the defect concentration after the thermal treatment of mrGO is also confirmed by the increase of I_D_/I_G_ and A_D_/A_G_ ratios (Table 1). However, the width of D band decreases after reduction and subsequent thermal treatment at 800 °C to a value as low as 89 cm^−1^. The narrowing of the D band correlates with an increase in sp^2^ content in the sample [39]. The lowest FWHM(D)s values are recorded for samples GOBA, GOBA/T and GOBA/G/T, indicating to expect the highest conductivity for these particular rGO samples. Moreover, it was demonstrated that the FWHM(D) is very sensitive to low energy structural defects (e.g., disorientations of the graphene layers) [40]. Therefore, similar conclusion as in the case of TG/DSC and SEM analysis (see Figure 1) can be drawn; reduction of GO in the melt of BA results in the less disordered rGO structure compared to the reduction of GO powder. However, though the FWHM(D) data indicate the increase in ordered layering of graphene during the thermal treatment up to 800 °C, SEM images show no substantial difference in morphology of both mrGO and trGO groups (see Figure S4 in Supplementary data). Surprisingly, analysis of correlation lengths Lα indicates that the high temperature treatment at 800 °C decreases the sp^2^-bonded carbon cluster size to ~18 nm for trGO. This effect may be due to the increase in defect concentration that results in smaller sp^2^ nanodomains than those in mrGO [41].

In order to verify the observed decrease of the correlation length, XRD analysis was performed. The XRD pattern are shown in Supplementary data section (Figure S5) and the data are summarized in Table 1. Values of the crystallite size are estimated using the width of (002) peak and the Scherrer formula [42]. The obtained results show, that GO/T product is composed of tiny rGO crystallites, which size of ~1.6 nm being the smallest among the other synthesized rGO samples. Moreover, the deviation of (100) peak position may show the distortion of crystal lattice in direction, which is parallel to (002) plane. The most significant deviation is observed for trGO samples. Most probably, this effect occurs due to the increased concentration of defects in trGO samples, which is determined from the analysis of D-band position in Raman spectra (Table 1). Finally, the XRD peak intensity ratio I_100_/I_002_ may be also used for the evaluation of crystal structure of graphene-based materials [43]. Different from I_D_/I_G_ ratio used in the analysis of Raman spectra, it represents the distortion of graphite-like crystal structure but not a single graphene layer. It was found that for GO/T, the I_100_/I_002_ ratio is higher in comparison with the majority mrGO and trGO samples. Consequently, the use of BA (except GOBA/G) is beneficial for the production of rGO of higher equivalence to graphite structure. The present data also suggests that the addition of glycerol significantly lowers the equivalence to graphite structure, when the product is heated up to 400 °C (GOBA/G). Further heating up to 800 °C diminishes the I_100_/I_002_ ratio (GOBA/G/T).

The porosity and specific surface area of electrode material used for the supercapacitor electrode is considered to be among the most important structural parameters. As mentioned in Introduction, the macroporous nature of graphene limits its volumetric energy density and the low packing density of graphene-based electrodes prevents its use in commercial applications [44]. The parameters obtained from nitrogen adsorption and desorption measurements are summarized in Table 2. The results show that, the value of S_BET_ and S_ext_ increases going from GO to mrGO. The GOBA/AA powder is characterized by the highest surface area (336 m^2^ g^–1^), however it is significantly lower that the theoretical surface area (2600 m^2^ g^–1^) reported for an individual graphene sheet [45]. Later, the surface area of samples gradually decreased (except GOBA/T) with increasing pre-treatment temperature but was much higher than that of pure GO. This divergence could be explained by the fact that N_2_ molecules are unable to penetrate the interlammellar space of obtained samples [46]. Moreover, the decrease of S_BET_, S_ext_ and total pore volume is the result of the textural reorganization which occurs during the treatment at higher temperatures [47]. In the case of GOBA sample, the surface area after additional heating increases and is related with the worsening of graphene layer ordering and increasing defect concentration I_D_/I_G_ = 1.48 (Table 1). It is worth noting, that the ratio S_ext_/S_BET_ of mrGO remains virtually unchanged after the thermal treatment (0.69 for GOBA and 0.64 for GOBA/T; 0.56 for GOBA/AA and 0.52 for GOBA/AA/T; 0.73 for GOBA/G and 0.72 for GOGA/G/T). This virtually constant S_ext_/S_BET_ ratio can be considered as an indicator showing the structural stability of a microparticle, where the micropores endure the impact of thermal treatment. 

**Figure 4:**
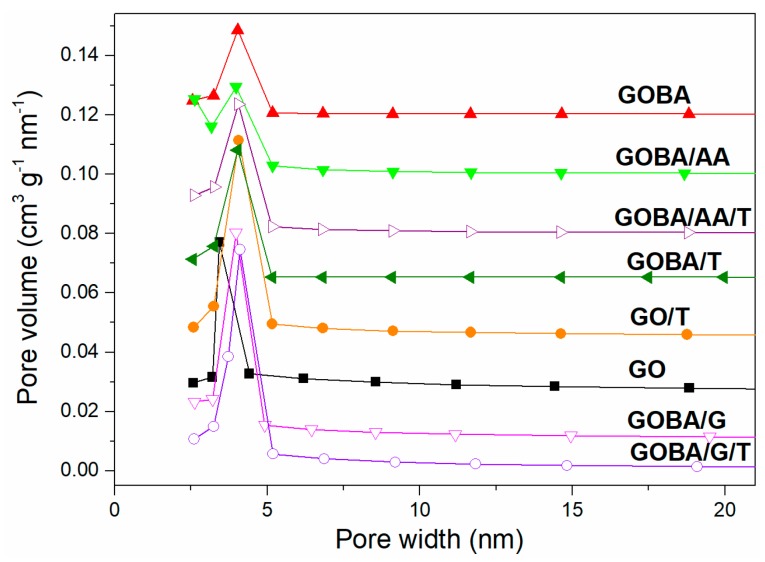
Pore size distribution of GO and rGO samples.

The pore size distribution curves of rGO samples are shown in Figure 4. They indicate presence of well-developed mesopores uniform in diameter (4 nm) for all rGO samples. The mesopores of GO are of slightly less in diameter (3.2 nm); this means that the modification is able to enlarge the interlayer distance between the graphene sheets, which remains virtually unchanged after the thermal treatment.

**Figure 5:**
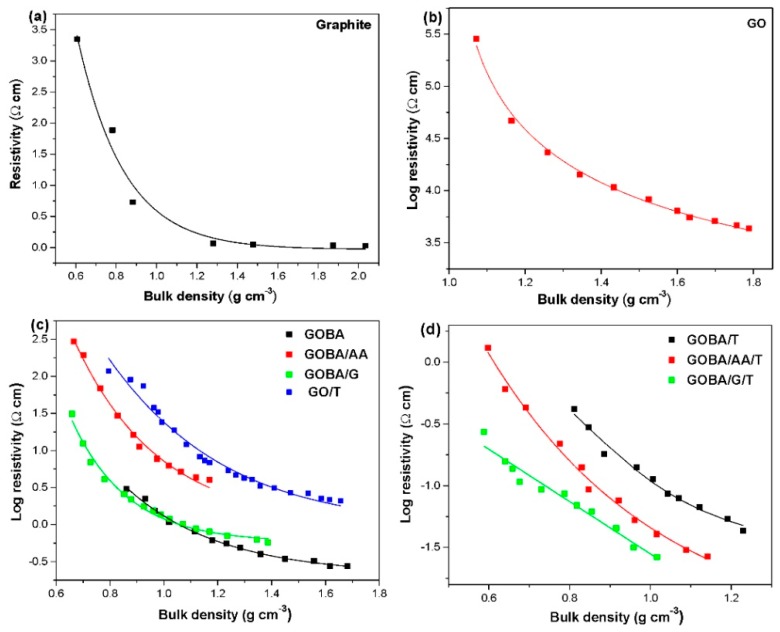
Specific resistivity dependence on bulk density for graphite (**a**), GO (**b**), GO/T and mrGO (**c**) and trGO (**d**).

Electrical conductivity of the synthesized rGO samples and their precursors vary in a wide range. The conductivity of powder specimen is dependent on the compression of the sample. Therefore, the impact of the bulk density increase on the resistivity of the sample was studied. The relationship between the electrical resistivity and the bulk density is shown in Figure 5a–d, which demonstrate that the electrical resistivity of the samples decreases when the bulk density increases. Under compression, the particles in the bulk material will be in contact closer with other adjacent particles; consequently, the density of bulk materials is higher. Following this, the higher density of bulk materials leads to the higher electrical conductivity due to more opportunities for the electrons to move across them. In contrast, a lower density of the bulk materials owns plenty of the gaps (the space between the adjacent particles), which strongly reduces mobility of electrons, thus, resulting in a lower electrical conductivity of the bulk materials. Resistivity of pristine graphite is dependent on the compression of the sample as well (Figure 4a); it varies in the range 0.03–3.4 Ω cm, approaching the theoretical values [21]. The resistivity of GO (shown in a logarithmic scale in (Figure 4b) is much higher than that of graphite due to the presence of abundant oxygen groups in the backbone of graphene sheet, which interrupt the sp^2^ π-electron network and consequently, GO becomes an insulator. The dependence of the resistivity on bulk density of GO/T, mrGO and trGO is shown using a logarithmic scale in Figure 4c,d respectively. As seen, the resistivity of rGO decreases significantly after the modification and thermal treatment. Moreover, the impact of compression on the resistivity of GO/T and mrGO is of asymptotic nature, while the same of trGO is much more linear. This divergence can be also attributed to the higher concentration of surface functional groups in GO/T and mrGO samples. Log resistivity (Ω cm) for trGO samples (Figure 5d) appear to be in the range from −0.55 to −1.59 for GOBA/G/T, from 0.13 to −1.58 for GOBA/C/T and from −0.55 to −1.59 for GOBA/T samples. These experimental observations indicate effective overall reduction of GO and restoration of the electronic structure of graphene planes. It has also been noted that the reducing agent type plays an important role in the electrical resistivity of GO. The GOBA/G/T sample is characterized by the highest electrical conductivity values, meanwhile GOBA/T — by the lowest ones.

### 3.3. Electrochemical Characterization

Characterization of the electrochemical behaviour of synthesized rGO samples was performed employing cyclic voltammetry. This characterization was used to evaluate double layer capacitance, C_dl_, of rGO samples. CVs in the double layer region reflect capacitance as proportion to a current density, especially for carbonaceous materials with oxygen containing functional groups [48]. CV curves obtained with the synthesized rGO in K_2_SO_4_ electrolyte are given in Supplementary data Figure S6. Three different loads of rGO (0.25, 0.50 and 1.0 mg mL^–1^) were used. A clear difference between trGO and mrGO samples was found: the non-faradaic current in the double layer region increased slowly at mrGO samples but the increase from 0.25 to 1.0 mg mL^–1^ rGO load was significant (more than 100%) for GOBA/AA/T and GOBA/G/T. The dependence of capacitance on rGO loads is given in Table 3. For all mrGO and trGO samples a 1.5−5.0 times increase in C_dl_ is observed, except GOBA/G/T, where this increase reached 14.5 times. As seen, the C_dl_ values of rGO samples are similar to reported elsewhere [49]. It must be noted that the response of GO/T was irreproducible; therefore, it was omitted from the further electrochemical analysis. 

**Figure 6:**
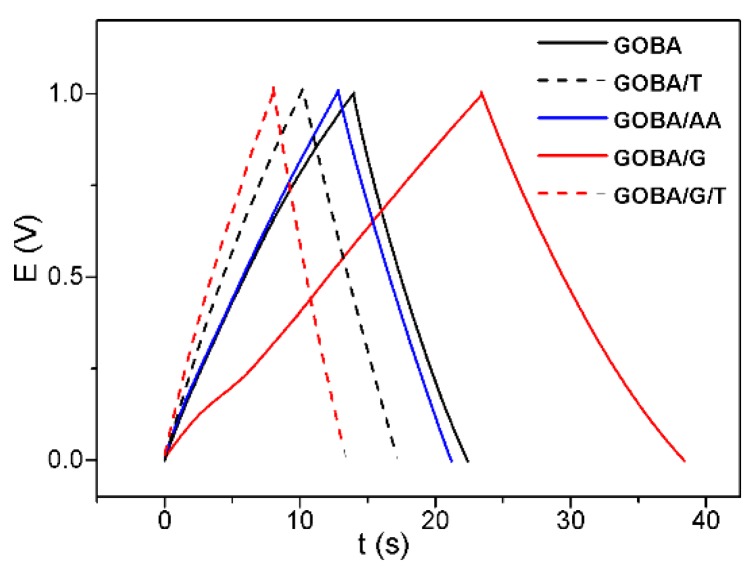
Charge discharge curves at the rGO electrodes: applied current 1.2 mA, electrolyte 0.1 M K_2_SO_4_, material load 2.4 mg.

In order to test the synthesized rGO samples closer to real supercapacitor conditions, charge/discharge curves were registered (Figure 6). The electrodes containing synthesized rGO were tested at constant current (1.2 mA) monitoring the potential dynamics. As seen in Figure 6, time in which potential reaches 1 V at applied current of 1.2 mA is different for all synthesized rGO samples. Since the potential of GOBA/AA/T electrode did not reach even the value of 1 V, data for GOBA/AA/T are not included in Figure 6. The fastest charge-discharge times were obtained for GOBA/G/T and GOBA/T, in particular, the charging times were 8 s and 10 s, respectively. The discharge time was the same as charging time for GOBA/G/T and faster than charging time for GOBA/T. These data suggest that trGO could be further optimised for capacitor or supercapacitor development. In the future, we intend to follow this direction and test the prepared samples in detail as electrode materials for supercapacitors. 

### 3.4. Impact of Structural Properties on Electrochemical Performance

As known, the electrochemical performance of supercapacitors depends on the structure and composition of electrode materials. Different authors, however, emphasize that the principle of optimization of electrode materials should be not only the improvement of the absolute structural characteristics but also a number of trade-offs [44]. For example, high electric conductivity of electrode material can lead to a better electrochemical performance, especially at high operating rates. On the other hand, high surface area of the electrode material is beneficial for the better capacitance. However, graphene with both a high surface area and high conductivity is difficult to synthesize. The optimal structure of rGO should be reached via a balanced trade-off using a controlled exfoliation of GO pristine material. A correlation between structure of rGO and their electrochemical performance should be considered as a threshold value, which is very sensitive to the whole set of structural parameters. Indeed, considerable differences observed in the electrochemical performance of rGO confirm this assumption.

One can find the positive correlation between the charge/discharge rate (Figure 6 and I_D_/I_G_ ratio in Raman spectra (Table 1). From this correlation, a conclusion can be drawn that higher defect concentration in synthesized rGO samples may improve electrochemical performance of rGO. Another pattern between the charge/discharge rate and Raman spectra is observed for ν_D_ and FWHM(D): the values for mrGO and trGO are concentrated in different zones and the values of charge/discharge rate are higher for trGO samples. From the discussion above it follows that this correlation can be associated with the increased concentration of frozen high-temperature defects. In some cases, these specific defects are beneficial for the fast ion transport in supercapacitors (GOBA/T and GOBA/G/T). The I_100_/I_002_ ratio obtained from the XRD patterns and the electrochemical performance of synthesized rGO show that samples with higher equivalence to the graphite crystal structure (lower I_100_/I_002_ value) possess higher charge/discharge rate. Surface chemistry of rGO also has a major influence on the performance of supercapacitor.

From the FTIR spectra it was determined that for mrGO the concentration of ketone C=O functional groups at vacancy sites decreases in the range GOBA/AA > GOBA > GOBA/G. After the thermal treatment at 800 °C these groups are almost removed from GOBA/T and GOBA/G/T, however, they remain in GOBA/AA/T.

**Figure 7:**
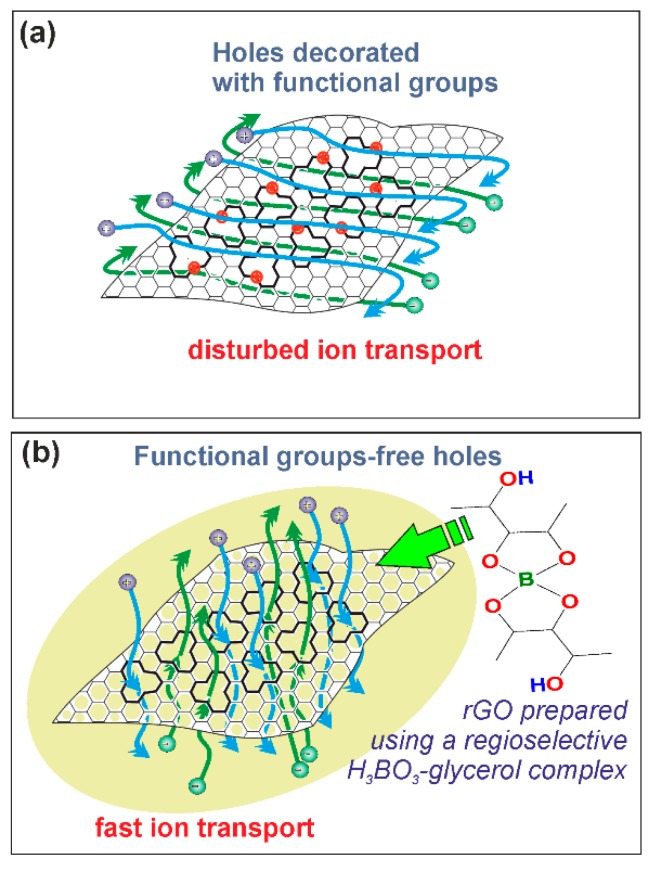
Formation of rGO structure without the presence of BA-glycerol catalyst (**a**) and in the presence of BA-glycerol catalyst (**b**). BA-glycerol catalyst prevents the formation of functional groups at the vacancy sites and facilitates the fast ion transport in electrode material.

Summarizing the results above it can be concluded that two main structural parameters impact on the electrochemical performance of synthesized rGO samples: the defects in the backbone structure of rGO and the ketone functional groups present at these defect sites. Increased concentration of functional group-free defects has a positive effect on the electrochemical performance, meanwhile, the increased concentration of ketone functional groups at the vacancy sites impair the supercapacitor characteristics. It was reported that at higher temperatures the defects in graphene backbone structure are able to merge into larger agglomerates (holes) [50]. These holes are beneficial for the fast transport of electrolyte ions [44]. Similarly, Tateishi et al. have reported that C–H defects produced from C=C bonds result in the huge specific weight capacitance for the reduced graphite oxide electrode [51]. Schematic illustration of the surface of rGO is given in Figure 7a,b.

A correlation between the porosity data and the electrochemical performance of rGO can also be observed. According to data represent in Table 2, it was determined that the higher values of S_ext_/S_BET_ are typical for rGO with reduced ratio of micropores and vice versa. Some authors emphasize that while the mesopores are beneficial for the easy approach of the electrolyte ions, micropores are able restrict the process of percolation [52]. Consequently, GOBA/G/T show the highest charge/discharge rate among the rGO samples due the highest values of S_ext_/S_BET_. While GOBA/AA and GOBA/AA/T characterize by the lowest values of S_ext_/S_BET_ and GOBA/AA/T exhibited the worst electrochemical performance in which potential of GOBA/AA/T electrode did not reach even the value of 1 V in charge/discharge experiment. In this connection, in Figure 4 and Table 2 one can see the highest mesopore volume fraction for GOBA/G and GOBA/G/T samples. Simultaneously, the GOBA/G/T sample is characterized by the highest electrical conductivity values (Figure 5d). The highest charge/discharge rates obtained for GOBA/G/T should be attributed to the operation of BA-glycerol chelate complex in regioselective epoxide ring-opening reaction, when the formation of ketone C=O surface functional groups at the vacancy sites is suppressed and further thermal treatment up to 800 °C leads to the formation of functional group-free holes in the backbone of rGO and optimal porosity which origin from 3D architecture of this rGO sample (Figure 7a,b). These observations should help to further develop of new and effective electrode materials for supercapacitors. 

Furthermore, the porosity well correlated with the double layer capacitance values, which is very important for energy storage. GOBA/G/T has the highest capacitance again due to the highest values of S_ext_/S_BET_ of this sample. Differently from charge-discharge test, the lowest capacitance was for GOBA, although the lowest porosity was for GOBA/AA. For capacitance, defects play extremely important role, which seemed stronger than the influence of mesopore size [53].

## 4. Conclusions

The reduction of GO in the H_3_BO_3_ melt was used, along with the employment of ascorbic acid and glycerol to reduce the disruption of graphene network. The TG/DSC results showed that the process of GO reduction in the melt of H_3_BO_3_ can be divided into two stages: (i) taking place in a wide range of temperatures (from 30 °C to 400 °C) and can be identified with the thermal reduction; (ii) coincided with the monotropic transitions between α-, β- and γ-metaboric acid phases and represents significant structural rearrangements of GO. 

FTIR analysis showed that the rGO samples treated with glycerol in the H_3_BO_3_ melt have a lower concentration of ketone C=O surface functional groups in comparison with the other ones. This effect was attributed to the catalytic performance of a BA-glycerol chelate complex, which is characterized by regioselectivity in epoxide ring-opening reactions and suppress the formation of C=O functional groups at vacancy sites. The Raman spectra confirmed that the rGO samples treated at 800 °C after melting in H_3_BO_3_ had higher defect concentration in comparison with those prepared by melting in H_3_BO_3_ at 400 °C. Supposedly, these were the holes produced at higher temperatures (800 °C) and frozen in the backbone structure of rGO.

Additionally, the correlation analysis between electrochemical performance of different rGO samples prepared in the H_3_BO_3_ melt and their structural parameters was carried out. Results show, that the defects in the backbone structure of rGO and absence of the ketone functional groups at these defect sites is of great importance to the electrochemical performance. Simultaneously, the GOBA/G/T was found the best electrode material for supercapacitor electrodes due to the increased concentration of functional group-free defects. Among others, GOBA/G/T sample showed the highest electrical conductivity values among all rGO samples. Moreover, optimal porosity of GOBA/G/T sample was confirmed by BET measurement.

Thus, our investigation demonstrates that GO reduction in the H_3_BO_3_ melt is an effective process for the rGO synthesis with less disrupted basal planes of graphene in comparison with those obtained by thermal reduction of GO powder. Further, this selective reduction of GO in the H_3_BO_3_ melt, enabled to produce rGO with better structural, electrical and electrochemical properties.

## Figures and Tables

**Table 1:** The results of Raman and X-Ray diffraction (XRD) analysis for rGO samples and their precursors.

Sample	Raman Analysis					XRD Characterization		
	D-band		I_D_/I_G_	A_D_/A_G_	L_α_ (nm)	Crystallite Size (nm)	2θ_100max_ (degrees)	I_100_/I_002_
	ν_D_ (cm^–1^)	FWHM (cm^–1^)						
GO	1344.7	133.5	1.12	1.62	24	6.961	42.25	0.381
GO/T	1342.6	137.8	0.96	1.76	22	1.577	42.96	0.170
GOBA	1336.1	111.2	1.10	1.98	19	3.078	42.97	0.099
GOBA/AA	1340.2	121.7	0.91	1.54	25	2.833	43.12	0.107
GOBA/G	1339.6	124.3	0.96	1.67	23	2.019	42.90	0.183
GOBA/T	1329.6	89.0	1.48	2.33	16	2.145	43.07	0.156
GOBA/AA/T	1329.4	114.4	1.21	2.10	18	2.455	43.00	0.141
GOBA/G/T	1329.9	97.1	1.29	2.10	18	2.934	43.09	0.135

**Table 2:** Textural characterization of rGO samples.

Sample	S_BET_ (m^2^ g^–1^)	S_ext_ (m^2^ g^–1^)	V_tot_ (cm^3^ g^–1^)	V_μ_ (cm^3^ g^–1^)	Average Pore Width (nm)
GO	46	41	0.17	0.00	15.13
GO/T	97	68	0.13	0.01	5.49
GOBA	66	46	0.07	0.01	3.99
GOBA/AA	336	188	0.20	0.07	2.41
GOBA/G	155	114	0.19	0.02	4.80
GOBA/T	107	68	0.09	0.02	3.42
GOBA/AA/T	229	118	0.15	0.05	5.68
GOBA/G/T	138	100	0.18	0.02	5.27

**Table 3:** Double layer capacitance dependence on rGO load.

Sample	C_dl_ for Different rGO Load (μF cm^–2^)		
	0.25 mg mL^–1^	0.50 mg mL^–1^	1.00 mg mL^–1^
GOBA	449	470	684
GOBA/AA	168	202	247
GOBA/G	107	183	260
GOBA/T	8.07	3.97	41.1
GOBA/AA/T	56.2	120	172
GOBA/G/T	208	794	3011

## Supplementary Materials

The following are available online at http://www.mdpi.com/2079-4991/8/11/889/s1, Table S1: Reaction conditions for the preparation of rGO samples, Figure S1: Typical current-voltage curves of the GOBA/G powder with bulk density of 0.659 g cm^−3^ (a); 0.853 g cm^−3^ (b); 0.988 g cm^−3^ (c); 1.169 g cm^−3^ (d); and 1.386 g cm^−3^ (e). The resistance of the sample was calculated form the slope of these curves. The linear shape of the curve was observed for all the samples in a voltage range ±1 V, Figure S2: TG (black line)/DSC (blue line) thermograms obtained in the systems composed of BA, GO, ascorbic acid and glycerol: (a) BA/G and precursor mixture of GOBA/G; (b) BA/C and precursor mixture of GOBA/AA; (c) trGO-GOBA/T; GOBA/AA/T and GOBA/G/T, Figure S3: Raman spectra of obtained samples. Raman spectra of pristine GO and GO/T (a). Raman spectra of mrGO samples (b). Raman spectra of trGO samples (c). Excitation wavelength is 632.8 nm (0.1 mW), Figure S4: SEM images of mrGO and trGO samples: GOBA (a); GOBA/AA (b); GOBA/G (c); GOBA/T (d); GOBA/AA/T (e); GOBA/G/T (f), Figure S5: XRD patterns of precursors and rGO powder (a), mrGO samples (b), trGO samples (c), Figure S6: CVs of the samples on PGE: GOBA (a); GOBA/T (b); GOBA/G (c); GOBA/G/T (d); GOBA/AA (e); and GOBA/AA/T (f). Concentrations are indicated on each graph. Electrolyte was 0.1 M K_2_SO_4_, potential scan rate 100 mV s^–1^.
