# Supplementary material for: Synthesis of Reduced Graphene Oxide with Adjustable Microstructure Using Regioselective Reduction in the Melt of Boric Acid: Relationship Between Structural Properties and Electrochemical Performance
Source: Nanomaterials (Basel). 2018 Nov 1;8(11):889. doi: 10.3390/nano8110889 (PMC6266621; doi:10.3390/nano8110889)
Supplement: Supplementary file 1 [file nanomaterials-08-00889-s001.zip › nanomaterials-378181-SI.pdf]

Supplementary data

Synthesis of reduced graphene oxide with adjustable microstructure using regioselective reduction in the melt of boric acid: relationship between structural properties and electrochemical performance

Justina Gaidukevič [1], Rasa Pauliukaitė [1,2], Gediminas Niaura [3,*], Ieva Matulaitienė [3], Olga Opuchovič [1], Aneta Radzevič [2], Gvidas Astromskas [4], Virginijus Bukauskas [4] and Jurgis Barkauskas [1]

1 Faculty of Chemistry and Geosciences, Vilnius University, Naugarduko str. 24, LT-03225 Vilnius, Lithuania; justina.gaidukevic@chf.vu.lt (J.G.); olga.opuchovic@chf.vu.lt (O.P.); jurgis.barkauskas@chf.vu.lt (J.B.)
2 Department of Nanoengineering, Center for Physical Sciences and Technology, Savanorių Ave. 231, LT-02300, Vilnius, Lithuania; pauliukaite@ftmc.lt (R.P.); aneta.radzevic@ftmc.lt (A.R.)
3 Department of Organic Chemistry, Center for Physical Sciences and Technology, Saulėtekio Ave. 3, LT-10257 Vilnius, Lithuania; ieva.matulaitiene@ftmc.lt (I.M.);
4 Department of Physical Technologies, Center for Physical Sciences and Technology, Saulėtekio Ave. 3, LT-10257 Vilnius, Lithuania; astromskas@ftmc.lt (G.A.); virginijus.bukauskas@ftmc.lt (V.B.)
* Correspondence: gediminas.niaura@ftmc.lt; Tel.: +370-65-264-9214

Table S1. Reaction conditions for the preparation of rGO samples.

Sample	Group	Precursor	Weight ratio	Preparation conditions	Yield (%)
GO/T		GO	-	Heating at 400 °C	-
GOBA	mrGO	GO + H_3BO_3	1 : 10	Melting at 400 °C	37.5
GOBA/AA		GO + H_3BO_3 + ascorbic acid	2 : 20 : 1	Melting at 400 °C	56.8
GOBA/G		GO + H_3BO_3 + glycerol	2 : 20 : 1	Melting at 400 °C	57.2
GOBA/T	trGO	GOBA	-	Annealing at 800 °C	71.3
GOBA/AA/T		GOBA/AA	-	Annealing at 800 °C	69.9
GOBA/G/T		GOBA/G	-	Annealing at 800 °C	74.0

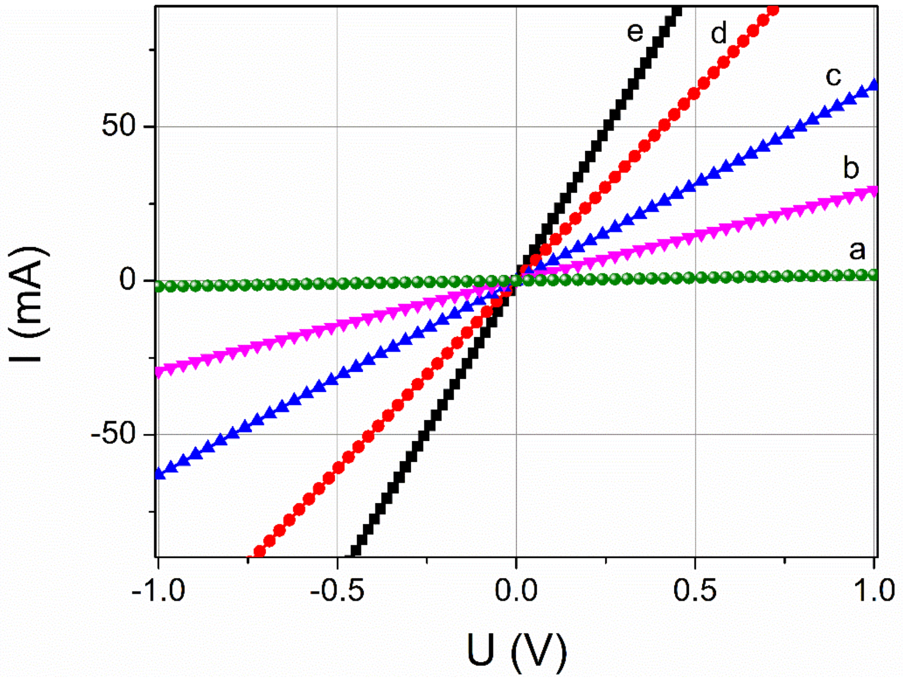

Figure S1. Typical current-voltage curves of the GOBA/G powder with bulk density of 0.659 g cm^{-3}(a); 0.853 g cm^{-3} (b); 0.988 g cm^{-3} (c); 1.169 g cm^{-3} (d); and 1.386 g cm^{-3} (e). The resistance of the sample was calculated form the slope these curves. The linear shape of the curve was observed for all the samples in a voltage range ±1 V.

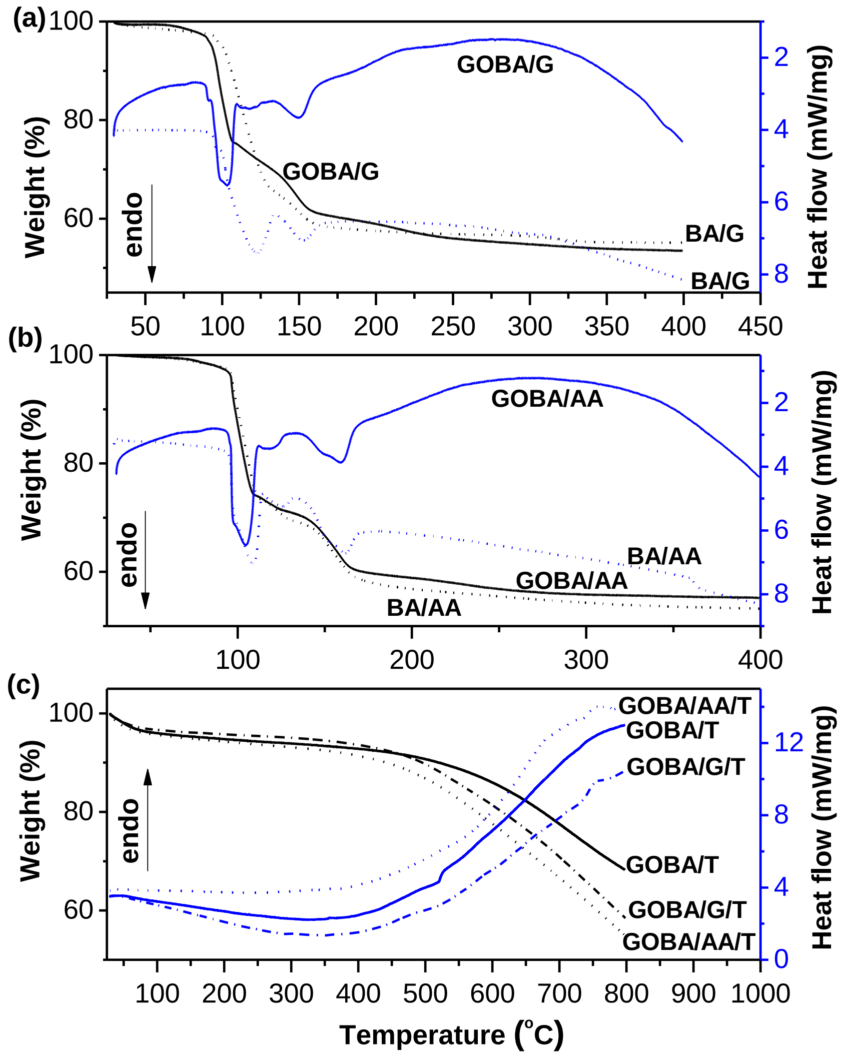

Figure S2. TG (black line)/DSC (blue line) thermograms obtained in the systems composed of BA, GO, ascorbic acid and glycerol: (a) BA/G and precursor mixture of GOBA/G; (b) BA/C and precursor mixture of GOBA/AA; (c) trGO - GOBA/T; GOBA/AA/T and GOBA/G/T.

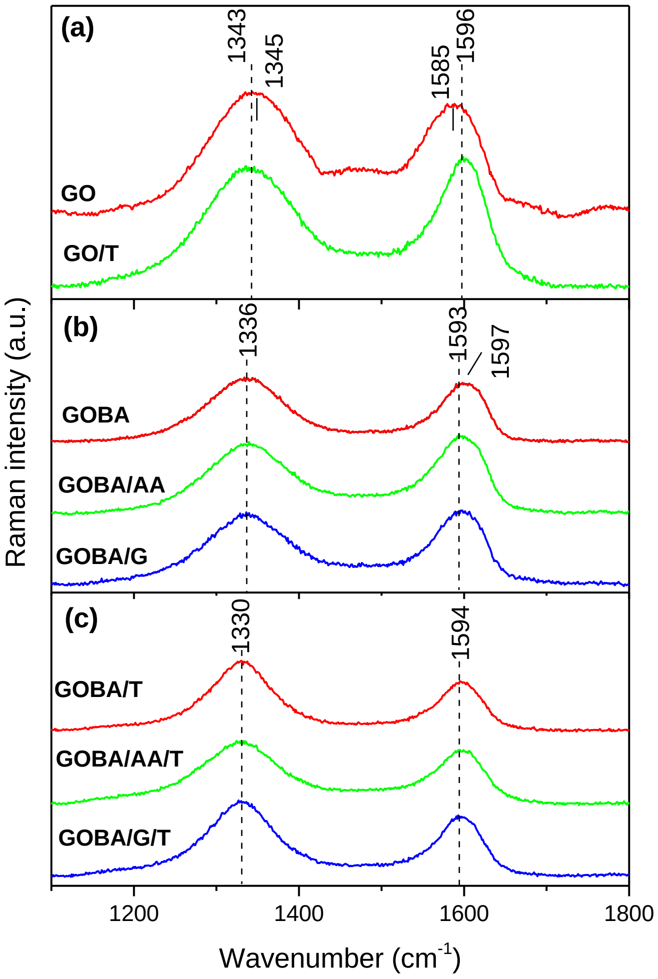

Figure S3. Raman spectra of obtained samples. Raman spectra of pristine GO and GO/T (a). Raman spectra of mrGO samples (b). Raman spectra of trGO samples (c). Excitation wavelength is 632.8 nm (0.1 mW).

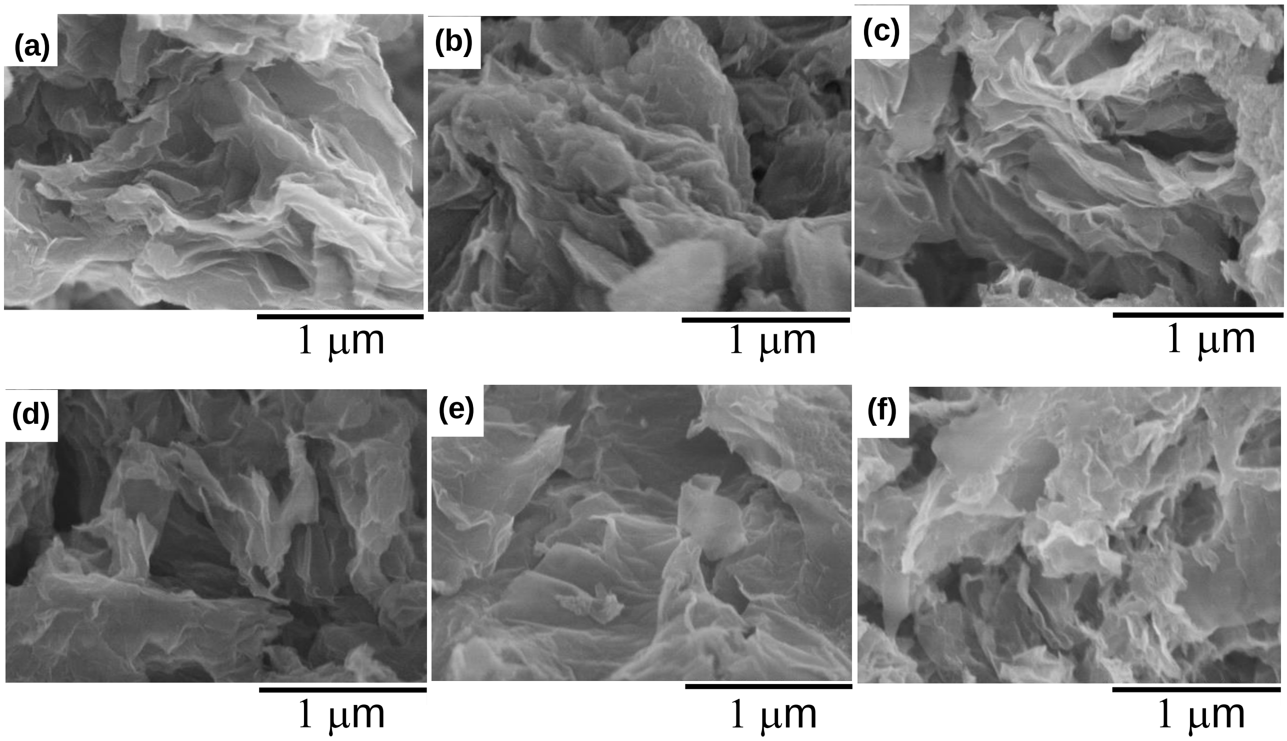

Figure S4. SEM images of mrGO and trGO samples: GOBA (a); GOBA/AA (b); GOBA/G (c); GOBA/T (d); GOBA/AA/T (e); GOBA/G/T (f).

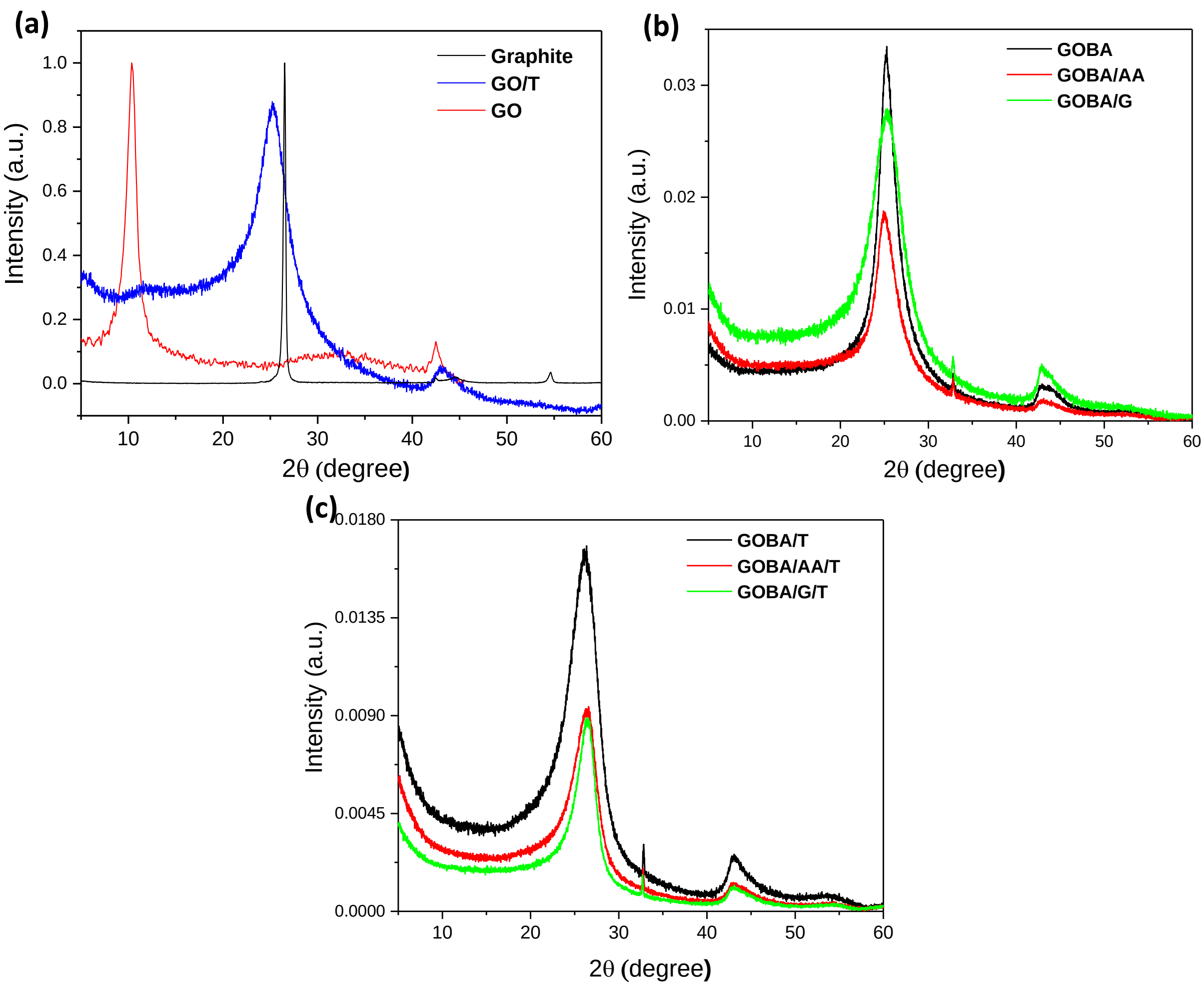

Figure S5. XRD patterns of precursors and rGO powder (a), mrGO samples (b), trGO samples (c).

The XRD pattern of graphite (Figure S4(a), black line) is compared to that in the PDF card 00-056-0159. A sharp peak at 2θ = 26.54° (002) as well as less intensive peaks at 2θ = 42.36° (100), 2θ = 44.56° (101) and 2θ = 55.66° (004) are present in the diffractogram of graphite.

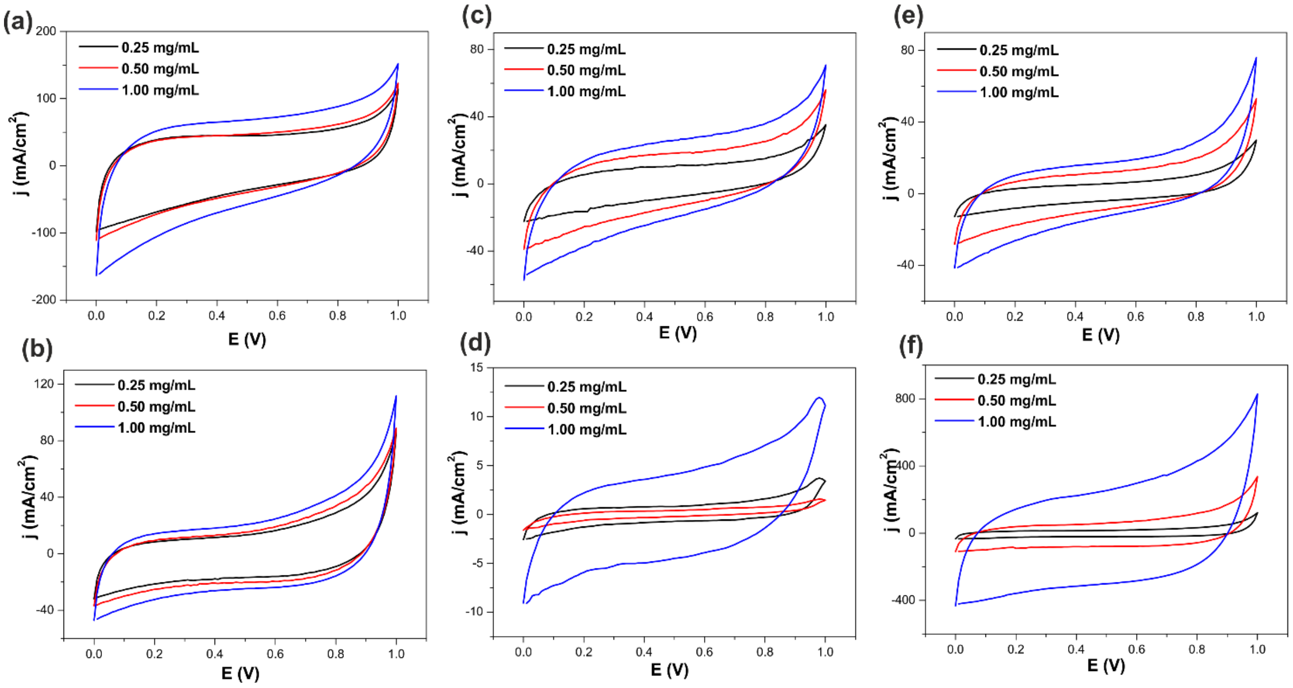

Figure S6. CVs of the samples on PGE: GOBA (a); GOBA/T (b); GOBA/G (c); GOBA/G/T (d); GOBA/AA (e); and GOBA/AA/T (f). Concentrations are indicated on each graph. Electrolyte was 0.1 M K_2SO_4, potential scan rate 100 mV s^{-1}.
